# Supplementary material for: Chromothripsis during telomere crisis is independent of NHEJ, and consistent with a replicative origin
Source: Genome Res. 2019 May;29(5):737–49. doi: 10.1101/gr.240705.118 (PMC6499312; doi:10.1101/gr.240705.118)
Supplement: Supplemental Material [file supp_gr.240705.118_Supplemental_file_1.zip › contigs/annotated_contigs/DB86/contig.2.DB86_length_616_mean_cov_8.76136363636.docx]

**DB86_length_616_mean_cov_8.76136363636**

```
ATTCTCCAGCAATAAGCCGTACACTCAGCTGTATCCAGTGGCTAGATAGTTCCTGAGAGATGTGTCCACAGTTCACTCAGAGTTCCTGT
 >chr11:88286481-88286816 + E=7e-190
TCCCCCTCATTTTCCTCTGATGTTCCTTCTGTGCCTTTTCTGCATTGTCATAATGTGTCTGCCTGAGTCAGAGCACAATGTCTGGTGTG

CCTGACTCTCACTAATGGGCCAAGTGTGGTCCTGCTCTCTTTAGCTTCTAATCAAGACTCCAGTGGGATCCAGAGTAAAAGTGAACAGG

CCACAGCATAGGGGAAAATTAAGATGGATGGTATAGCACAGCAGGAGCTTGTTTATCCCTGA|GGAATA|GCATGTGACCATGTTTGGG
                                                               >chr11:88287581-88287824
GGCCATCATCTGGCTACTAAAGAGGACTTCCAGGTTGATATTATTCTATCCTATTACTTCCCTTGTTTTATTCATGCTATTGAAGATGT
 + E=3e-103
AATAAGATTTTTTGTTTTTTGTTTAATTTAGCTTTTGTTTTTACAGCAATTCTACATGATGATGCTGAGATAATCGCTGATTTATATCC

CAAGTTTTTTTCTAATTCCCTGTTAATTAACTTTGTTACTGCATCT|TTCTCTGGTGCAGTTCTTTGATTGGTCATGTTTGTACG
```
